# Robotic-Assisted Resection of High-Grade Primary Retroperitoneal Mucinous Cystadenocarcinoma: A Case Report and Literature Review

**DOI:** 10.7759/cureus.110558

**Published:** 2026-06-09

**Authors:** Shivansh Pandey, Ayush Patel, Mohamed Aboelmaaty, Naser Mubarak, Jared Johnson, Hamed Ahmadi

**Affiliations:** 1 Urology, University of Minnesota, Minneapolis, USA; 2 Medicine, University of Central Florida College of Medicine, Orlando, USA; 3 Public Health, University of Pennsylvania, Philadelphia, USA

**Keywords:** cystic retroperitoneal mass, oncologic urology, primary retroperitoneal mucinous cystadenocarcinoma, urology, urology surgery

## Abstract

Primary retroperitoneal mucinous cystadenocarcinoma (PRMC) is an exceptionally rare malignancy of uncertain histogenesis, with fewer than 110 cases reported in the literature since its initial characterization in 1965. PRMC typically presents as a large cystic retroperitoneal mass, but diagnosis remains challenging due to nonspecific imaging features and lack of biomarkers. Surgical resection remains the cornerstone of management, while the role of adjuvant therapies remains controversial. We present the case of a 46-year-old woman with high-grade PRMC successfully managed with robotic-assisted resection, to the best of our knowledge, the first reported malignant PRMC resection via this technique. We supplement this report with a literature review of clinicopathological features, proposed histogenesis, and therapeutic outcomes.

## Introduction

Primary retroperitoneal mucinous cystadenocarcinoma (PRMC) is extremely rare, with <110 cases described since 1965 [1]. Diagnostic ambiguity arises from nonspecific radiographic findings and the absence of reliable biomarkers. The standard of care is complete surgical resection without rupture. The role of adjuvant chemotherapy is not well established [2]. We present a novel case of high-grade PRMC treated with robotic-assisted resection, which, to our knowledge, represents the first documented robotic resection of a malignant PRMC, along with a focused literature review.

Primary retroperitoneal mucinous tumors are generally described across a histologic spectrum that includes benign mucinous cystadenoma, borderline mucinous tumor, and mucinous cystadenocarcinoma. Distinguishing primary malignant retroperitoneal disease from metastatic mucinous adenocarcinoma is essential because gastrointestinal, pancreaticobiliary, appendiceal, ovarian, and renal primaries may mimic PRMC clinically, radiographically, and histologically.

## Case presentation

A 46-year-old woman presented in March 2024 with three weeks of sharp left upper quadrant abdominal pain, worse at night and when lying on her left side. She denied fevers or urinary symptoms. Family history was notable for hypertension (mother) and deep vein thrombosis (father). Physical exam showed left costovertebral angle and left upper quadrant (LUQ) tenderness.

Diagnostic workup

Laboratory evaluation was unremarkable except for mild hyperglycemia. CT imaging (April 2024) showed a 5.3 cm lesion along the left kidney (Figure 1).

**Figure 1:**
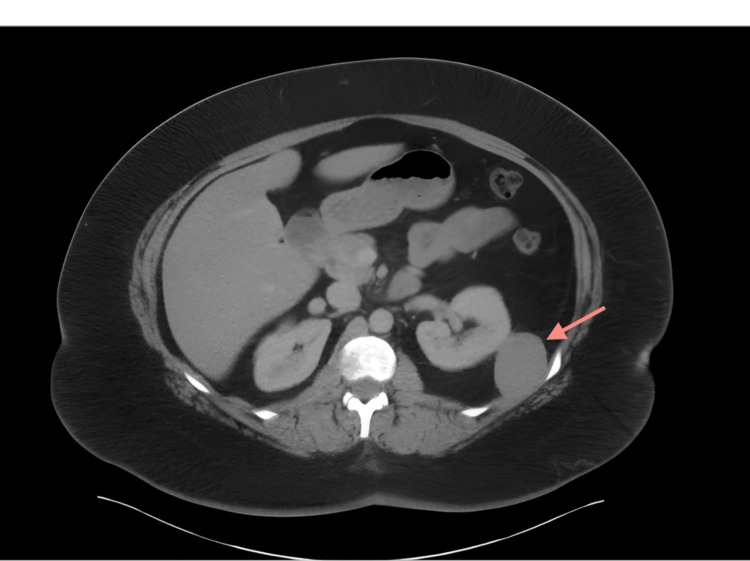
CT showing a 5.3 cm lesion along the lateral margin of the left kidney, initially favored to represent a benign hyperdense exophytic cyst

Ultrasound confirmed a complex cystic structure (5.6 cm) (Figure 2).

**Figure 2:**
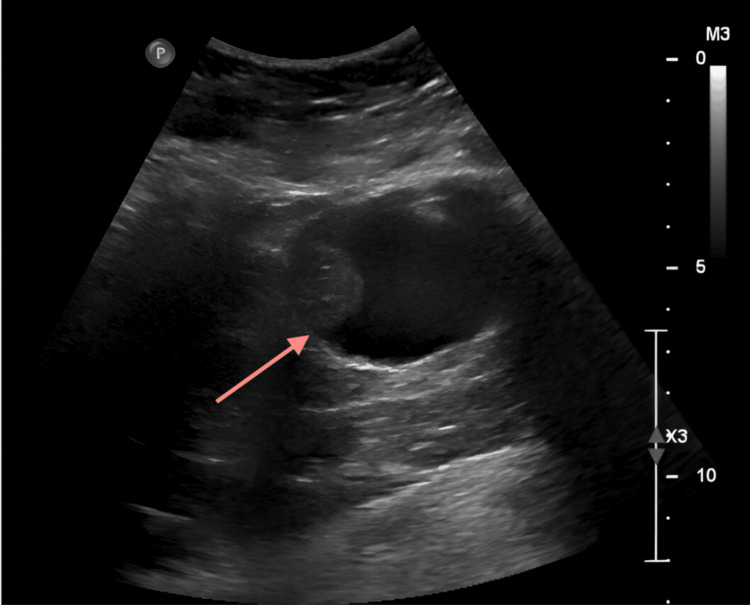
Ultrasound showing a 5.6 cm complex cystic lesion along the lateral mid left kidney, initially interpreted as a complex cystic renal lesion with internal debris or blood products

MRI (May 2024) suggested a hemorrhagic cyst, though internal enhancement raised suspicion for cystic renal cell carcinoma (Figure 3). A robotic partial nephrectomy was planned.

**Figure 3:**
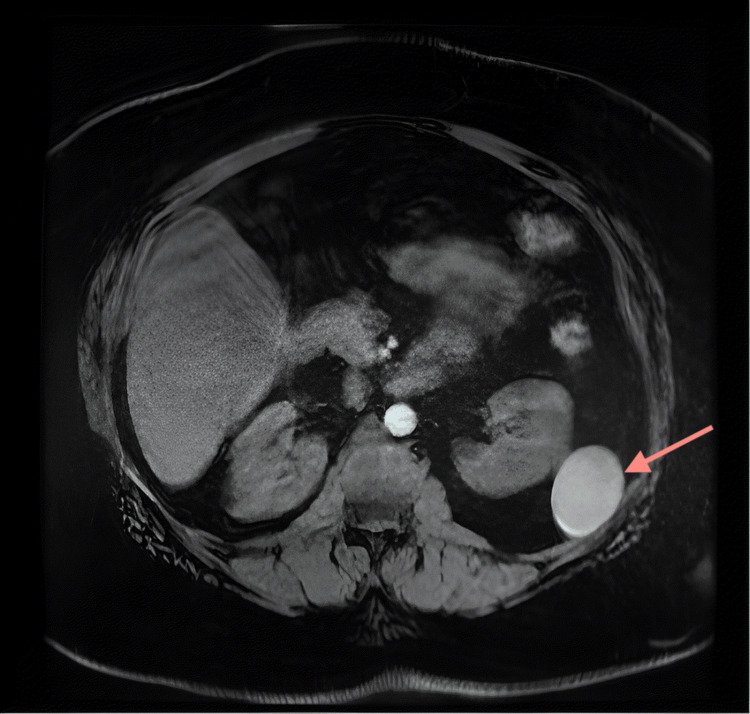
MRI showing a 5.0 cm lesion along the posterolateral left kidney with internal T1 shortening and faint internal enhancement, raising concern for cystic renal cell carcinoma

Surgical findings

In July 2024, the patient underwent robot-assisted laparoscopic excision of a left perirenal mass. The patient was placed in a modified flank position. Pneumoperitoneum was established using a Veress needle, followed by placement of a camera port, three 8-mm robotic ports, and a 12-mm assistant port. The colon was reflected medially to expose the anterior surface of Gerota’s fascia. The lesion was identified lateral to the left kidney within the perinephric/retroperitoneal space. Intraoperatively, there was no clear attachment to the kidney itself; rather, the mass appeared more adherent to the lateral abdominal wall musculature and adjacent perinephric fat. Multiple parasitic vessels feeding the lesion were controlled using a vessel sealer, bipolar cautery, and sharp dissection (Figure 4). Care was taken to avoid diaphragmatic injury. After circumferential mobilization, hemostatic agents were applied to the resection bed with excellent hemostasis. The specimen was placed in an Endo Catch (Medtronic, Minneapolis, MN, USA) bag and extracted through a slightly extended 12-mm assistant port site. Estimated blood loss was 50 mL, and there were no intraoperative complications.

**Figure 4:**
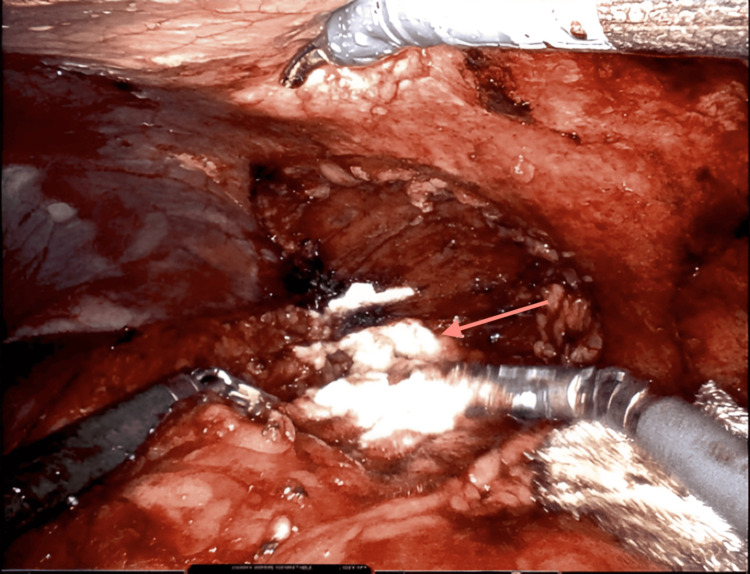
Intraoperative robotic view of the left perirenal/retroperitoneal mass The arrow identifies the operative target corresponding to the left perirenal mass. Intraoperatively, the lesion was located lateral to the left kidney, had no clear attachment to the kidney itself, and appeared more adherent to the lateral abdominal wall musculature and adjacent perinephric fat.

Pathology

Final pathology demonstrated a 5.8 cm high-grade mucinous adenocarcinoma with cystic and papillary growth patterns intersected by fibrous septations containing ovarian-type stroma. No renal tissue was identified, and the tumor appeared to be exclusively within the retroperitoneum, supporting a diagnosis of primary retroperitoneal mucinous cystadenocarcinoma. Margins were negative for malignancy, although carcinoma was focally less than 0.1 cm from the margin. Pathology also noted evidence of prior cyst rupture, warranting close surveillance. Tumor necrosis was present, and lymphovascular invasion was not identified.

Immunohistochemistry showed diffuse positivity for PAX8, HNF-1β, and CK7, with patchy CDX2 reactivity. CK20 and Napsin A were negative. SF1, WT1, and ER highlighted the ovarian-type stroma in a patchy distribution. Additional staining showed retained FH and negative 2SC, arguing against FH-deficient renal cell carcinoma. Molecular testing identified pathogenic KRAS p.G12D and TP53 p.R306* alterations, with low tumor mutational burden. A separate fusion panel was negative for therapeutically or diagnostically relevant rearrangements.

Outcome

Postoperative recovery was uneventful; the patient was discharged on POD1. Surveillance CT scans at approximately 3, 6, 12, and 18 months showed no evidence of recurrence or metastasis.

## Discussion

We performed a literature review using PubMed through June 2026. Search terms included “primary retroperitoneal mucinous cystadenocarcinoma,” “primary retroperitoneal mucinous tumor,” “retroperitoneal mucinous cystic neoplasm,” “PRMC,” “robotic,” “robot-assisted,” “laparoscopic,” and “resection.” We included literature describing primary retroperitoneal mucinous tumors and excluded clearly metastatic mucinous tumors from ovarian, gastrointestinal, pancreaticobiliary, appendiceal, or other primary sites. Our review identified 108 previously reported PRMC cases since 1965 [1]. The present case represents the 109th reported case of PRMC and, to our knowledge, the first documented robotic resection of a malignant PRMC. Of the 109 PRMC cases, only 11 have been reported in males, suggestive of a predominantly female pathology [1].

The exact origin of PRMC remains a subject of ongoing inquiry, with several theories proposed to explain its histogenesis and pathogenesis. These theories include (1) heterotopic ovarian tissue, supported by PRMC’s estrogen receptor positivity and similarity to ovarian mucinous cystadenomas, (2) monodermal variant of teratomas from displaced germ cells, (3) embryonal urogenital remains, (4) intestinal duplication, and (5) coelomic metaplasia [3]. Previous case reports have described cystic tumors surrounded by smooth muscle fibers and intestinal-like epithelium, providing support for some of these theories [4]. However, the coelomic metaplasia hypothesis has garnered the most support. During embryonic development, fragments of coelomic epithelium may become entrapped within the retroperitoneal space. These isolated epithelial remnants could potentially form inclusion cysts, which may later serve as the origin for retroperitoneal tumors when stimulated by steroids and inflammation [5].

The preoperative diagnosis of PRMC presents significant challenges due to its nonspecific imaging characteristics and the limited utility of tumor markers. PRMC typically manifests as a cystic mass within the retroperitoneal space, making it difficult to differentiate from other benign or malignant neoplasms based solely on imaging studies. Radiographic findings may include thickening and calcification of the cyst wall or mural nodules that suggest malignancy [6]. Similarly, in our case, a hyperdense lesion with internal enhancement was identified. Our case and previous reports in the literature highlight a common diagnostic challenge: the initial suspicion of renal cell carcinoma. This misdiagnosis may persist until intraoperative observations reveal that the tumor is separate from the kidney and subsequent pathological examination confirms the absence of renal tissue [7]. This pattern suggests the definitive diagnosis of PRMC is heavily dependent on histopathological analysis. Preoperative imaging and clinical presentation often fail to provide conclusive evidence, making it difficult to distinguish PRMC from other retroperitoneal masses. Unlike some other malignancies, PRMC does not consistently produce specific biomarkers that could aid in its preoperative identification. As a result, tumor markers such as carcinoembryonic antigen (CEA), cancer antigen (CA) 19-9, and CA-125 have inconsistent expression patterns [7]. Furthermore, in cases where these markers are initially elevated, their levels often normalize following surgical resection of the tumor.

Open and laparoscopic surgical approaches have been previously utilized in the treatment of PRMC [1,3]. Open surgery has traditionally provided broad exposure of the tumor and surrounding tissues, reducing the risk of incomplete resection in complex cases. This approach may be favored when tumor size, location, suspected invasion, adhesions, or concern for rupture make minimally invasive resection less appropriate. Laparoscopic surgery offers a less invasive alternative but still carries the risk of intraoperative cyst rupture, potentially leading to tumor content spillage and increased recurrence risk [1].

Robotic resection presents an advanced approach, combining the benefits of minimally invasive surgery with enhanced precision and control [8]. In this case, the tumor was located lateral to the left kidney, with no clear attachment to the kidney itself, and appeared more adherent to the lateral abdominal wall musculature and adjacent perinephric fat. The robotic approach enabled circumferential mobilization of the lesion and control of parasitic feeding vessels using vessel sealer, bipolar cautery, and sharp dissection. The specimen was removed in an Endo Catch (Medtronic) bag. Prior robotic reports have described non-malignant or borderline retroperitoneal mucinous tumors. Rodman et al. reported robotic removal of a retroperitoneal mucinous cystic neoplasm, Mudhher et al. reported robotic management of a benign primary retroperitoneal mucinous cystadenoma, and Zhang et al. reported borderline primary retroperitoneal mucinous cystic tumors, including a robot-assisted case that recurred after intraoperative cyst opening [9-11]. In contrast, the present case involved high-grade malignant PRMC. To our knowledge, this is the first documented robotic resection of a malignant PRMC. The absence of documented intraoperative rupture or spillage, complete circumferential mobilization, and Endo Catch (Medtronic) bag extraction are important oncologic details, particularly given the recurrence risk associated with cyst rupture.

Given the rarity of PRMC, there are no established guidelines for adjuvant chemotherapy after complete resection. In this case, surveillance was pursued because the tumor was completely resected with negative margins, and postoperative imaging showed no regional or distant metastatic disease. However, the decision for surveillance was made in the context of close follow-up rather than presumed absence of risk, as the tumor demonstrated high-grade histology, pathogenic KRAS p.G12D and TP53 p.R306* alterations, a focally close margin of less than 0.1 cm, and pathologic evidence of prior cyst rupture. These features supported close radiographic surveillance. No formal multidisciplinary tumor board review or separate medical oncology recommendation was documented in the available chart.

This report has several limitations. Histopathology images were not available for inclusion, limiting visual documentation of the tumor’s microscopic features. Serum tumor markers, including CEA, CA 19-9, and CA-125, were not obtained preoperatively or postoperatively. In addition, the specific robotic platform was not documented in the available operative record.

## Conclusions

PRMC is an exceedingly rare malignancy with diagnostic ambiguity and unclear optimal management. Complete surgical resection remains the standard of care. Robotic-assisted resection may represent a safe and effective minimally invasive alternative in select cases. Continued case reporting is essential to define prognostic factors and long-term outcomes.
